# Adult spinal cord diffuse midline glioma, H3 K27-altered mimics symptoms of central nervous system infection: a case report

**DOI:** 10.3389/fneur.2023.1097157

**Published:** 2023-06-15

**Authors:** Xue Chen, Yi Li, Hui Bu, YueLi Zou, JunYing He, Hu Liu

**Affiliations:** Department of Neurology, The Second Hospital of Hebei Medical University, Shijiazhuang, China

**Keywords:** adult, spinal cord neoplasms, central nervous system infection, case report, diffuse midline glioma, H3 K27-altered

## Abstract

Diffuse midline gliomas, H3 K27-altered are infiltrative growth gliomas with histone H3K27M mutations. This glioma is more common in the pediatric population, and the prognosis is usually poor. We report a case of diffuse midline gliomas, H3 K27-altered in an adult patient that mimicked symptoms of central nervous system infection. The patient was admitted due to double vision for 2 months and paroxysmal unconsciousness for 6 days. Initially, lumbar puncture showed persistent high intracranial pressure, high protein, and low chlorine. Magnetic resonance imaging showed diffuse thickening and enhancement of meninges and spinal meninges, and later, fever occurred. The initial diagnosis was meningitis. We suspected central nervous system infection, so we started anti-infection treatment, but the treatment was ineffective. The patient's condition gradually worsened, with lower limb weakness and even the consciousness became unclear. A repeat magnetic resonance imaging and positron emission tomography–computed tomography scan showed space-occupying lesions in the spinal cord, which was considered a tumor. Following neurosurgery, pathological tests identified the tumor as diffuse midline gliomas, H3 K27-altered. The patient was recommended for radiotherapy and temozolomide chemotherapy. The patient's condition improved after chemotherapy treatment, and he survived for an additional 6 months. Our case shows that diagnosing diffuse midline gliomas, H3 K27-altered in the central nervous system is complex and can be confused with the clinical characteristics of central nervous system infection. Therefore, clinicians should pay attention to such diseases to avoid misdiagnosis.

## Introduction

In 2016, the World Health Organization (WHO) classified diffuse midline glioma (DMG) H3K27M mutant as a new and independent subtype of diffuse astrocytoma of the central nervous system (CNS). This was updated to DMG, H3 K27-altered, in the 2021 WHO Classification (1). It is defined as a high-grade (grade IV) glioma with diffuse midline invasion. These gliomas are characterized by astrocyte differentiation and K27M mutation either in H3F3A or, more rarely, in HISTlH3B/C genes (2). The tumors are often located in midline structures (the brainstem, thalamus, and spinal cord) (1) and rarely occur in the third ventricle, hypothalamus, pineal gland, and cerebellum. It may be accompanied by leptomeningeal dissemination (3). It occurs predominantly in children and rarely in adults, but spinal cord sites mainly occur in adults (4). For diffuse gliomas located in midline structures, the incidence of H3K27M mutations is ~80% in children and 15–60% in adults (5). The prognosis of this disease is poor, and studies show that the 2-year survival rate is <10% (6). In this report, we present a case of adult DMG, H3 K27-altered that initially presented with persistent high intracranial pressure and diffused meningeal and spinal meninges enhancement. It was initially misdiagnosed as a CNS infection.

## Case presentation

**Figure 1:**
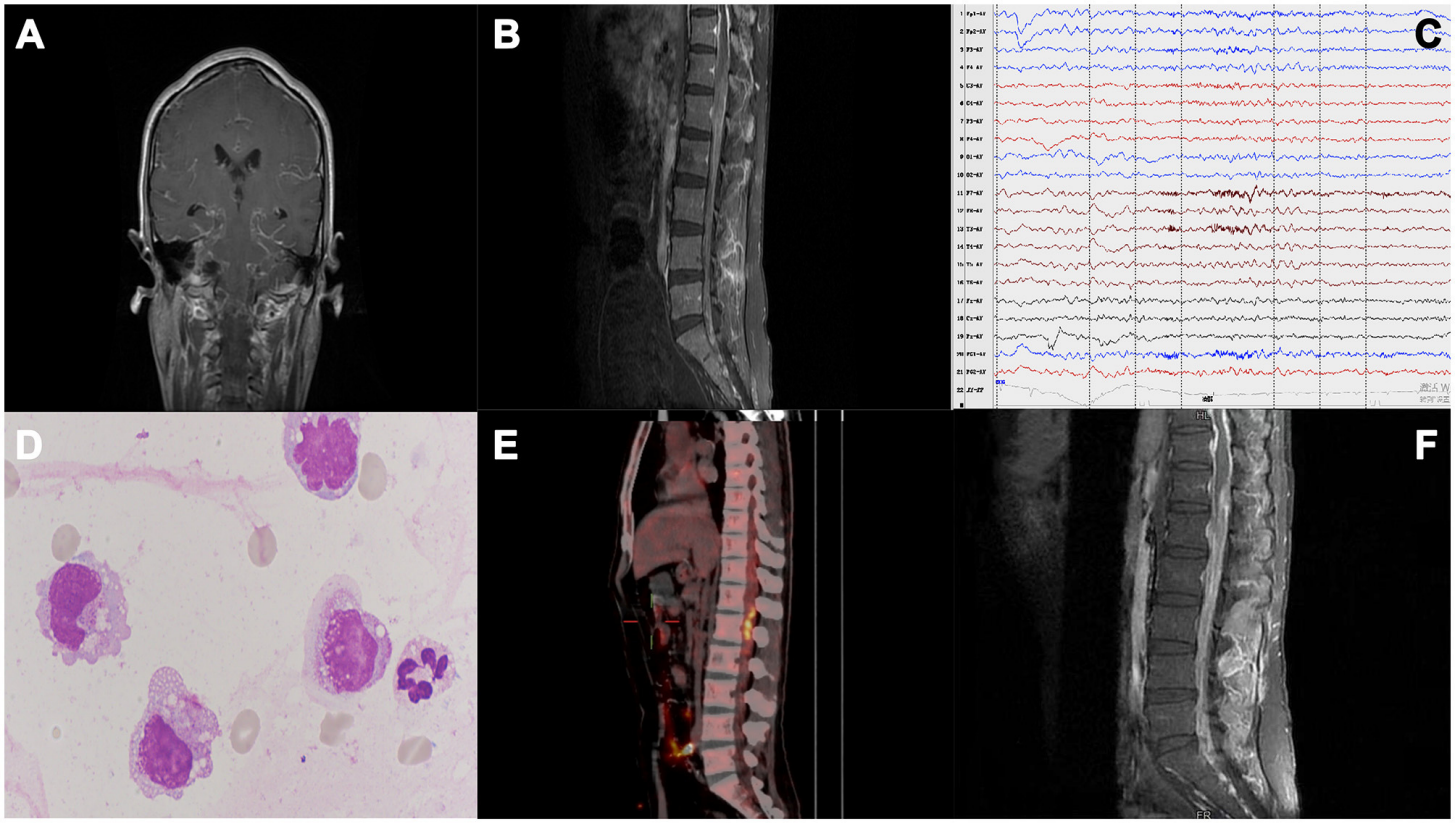
**(A)** Cranial MRI scan showing diffuse meningeal enhancement. **(B)** Thoracic and lumbar spinal cord MRI showed extensive thickening and enhancement of the spinal dural of the thoracolumbar and sacral canal, especially around the end of the spinal cord. **(C)** Electroencephalogram showing moderately abnormal electroencephalogram; background activity was slow, and numerous θ slow waves; frontal and temporal regions showed mixed slow wave firing, especially on the left side. **(D)** CSF cytology shows an increased number of activated monocytes. **(E)** PET-CT shows multiple hypermetabolic areas scattered in the spinal canal at the level of the T2-L2 vertebral body, thus indicating the possibility of intramedullary tumors with intraspinal metastasis. **(F)** Lumbar MRI: 1. Extensive thickening and enhancement of the dura mater of the thoracolumbar and included sacral canals, especially around the end of the spinal cord, which did not change significantly from 1st MRI. 2. Abnormal signals in the spinal cord at about the T12–L1 vertebra level.

A 32-year-old man was admitted to a local hospital with paroxysmal episodes of unconsciousness for 6 days and had convulsions 2 days ago. The patient also reported suffering from diplopia for 2 months, accompanied by headache, nausea, and vomiting. Lumbar puncture showed a cerebrospinal fluid (CSF) pressure of >330 mmH_2_O and protein level at 440 mg/dL, CSF WBC count was 1 × 10^6^/L, and CSF color was yellow. The patient was diagnosed with encephalitis and was given antiviral and anti-inflammatory treatments, but there was no significant improvement in his condition. He was later transferred to our hospital for further treatment. His past medical history showed “lumbar disk herniation” for more than 10 years. In the recent 2 months, his symptoms had worsened, accompanied by discomfort in the right lower limb. Respiratory infection and diarrhea were not indicated in the medical history before this disease. Family history showed that the patient's maternal grandfather had a history of tuberculosis 30 years ago, which was cured. The patient's sister had died of intracranial glioma at 6 years of age, and a pathological test was not performed. At the time of admission, the patient was conscious and showed fluent speech, and muscle strength of both upper limbs was grade V and of lower limbs was grade V minus. The Babinski sign was negative bilaterally. Both T-SPOT and PPD tests were positive. A Cranial magnetic resonance imaging (MRI) scan showed diffuse meningeal enhancement caused by infection (Figure 1A). The patient was therefore diagnosed to have tuberculous meningitis. Following admission, he was treated with mannitol and glycerin fructose to reduce intracranial pressure, and anti-tuberculosis treatment was started.

During hospitalization, the patient complained of lower back pain and increased weakness in both lower limbs. On the 7th day, the patient had seizures, and his body temperature increased to 38°C. The patient still has headaches and vomiting. Physical examination showed that the patient was somnolent but arousable to stimulus, had limited abduction of both eyes, had sensitivity to pupillary light reflexes, showed grade IV muscle strength in both the upper limbs, had a stiff neck, showed grade II muscle strength in both the lower limbs, and had hypesthesia of both lower limbs, and the bilateral Babinski sign showed all toes were downgoing. MRI scans of the thoracic and lumbar spinal cord were again performed on the 8th day following hospitalization. The results showed extensive thickening and enhancement of the thoracolumbar spinal meninges (Figure 1B). Electroencephalogram showed increased slow waves in frontal and temporal lobes (Figure 1C). Two lumbar punctures were performed on day 2 and day 7 of hospitalization, and the results showed a significant increase in intracranial pressure and protein level and a decrease in chloride level (Table 1). Acid fast stain and tuberculosis cultures were negative. Though the anti-tuberculosis treatment showed no improvement in the patient's condition, the treatment was continued as his body temperature remained high, up to 38.5°C. The next-generation sequencing of CSF for detecting associated infections and three subsequent lumbar punctures provided no additional diagnostic information (Table 1). After 18 days of anti-infective treatment, the patient's symptoms of high intracranial pressure did not improve, but his body temperature returned to a normal level. The intracranial pressure remained high, with high protein and low chlorine levels. However, we noticed that there was no significant increase in the white blood cell count, and the cytology of the patient's cerebrospinal fluid showed an increase in the number of activated monocytes while malignant cells were not detected (Figure 1D). These results lead us to consider the presence of tumors in the spinal cord. A positron emission tomography-computed tomography (PET-CT) scan was performed, and the results showed multiple hypermetabolic areas scattered in the spinal canal at the level of the T2-L2 vertebral body, thus indicating the possibility of intramedullary tumors with intraspinal metastasis (Figure 1E).

**Table 1:** Cerebrospinal fluid routine biochemistry and cytology.

**Date parameters**	**Before admission**	**Day 2**	**Day 7**	**Day 21**	**Day 23**
Intracranial pressure	>330 mmH_2_O	>330 mmH_2_O	>330 mmH_2_O	>330 mmH_2_O	>330 mmH_2_O
White blood cells (0–8)	2	96	7	6	1
Protein (8–32 mg/dL)	440	367	343	367	353
Glucose (2.5–4.5 mmol/L)	3.68	3.29	3.03	3.24	4.02
Chlorine (120–132 mmol/L)	107	93.2	92.5	95.6	102.1
Cytology	–	Abnormal cerebrospinal fluid cytology with mixed cytological reaction. 36% lymphocytes, 31% neutrophils, activated monocytes: 9%	Abnormal CSF cytology, activated monocytosis, 9% neutrophils seen, activated monocytes 59%	–	Abnormal cerebrospinal fluid cytology with mixed cytological reaction. Lymphocytes 36%, neutrophils 28%, activated monocytes 20%

**Figure 2:**
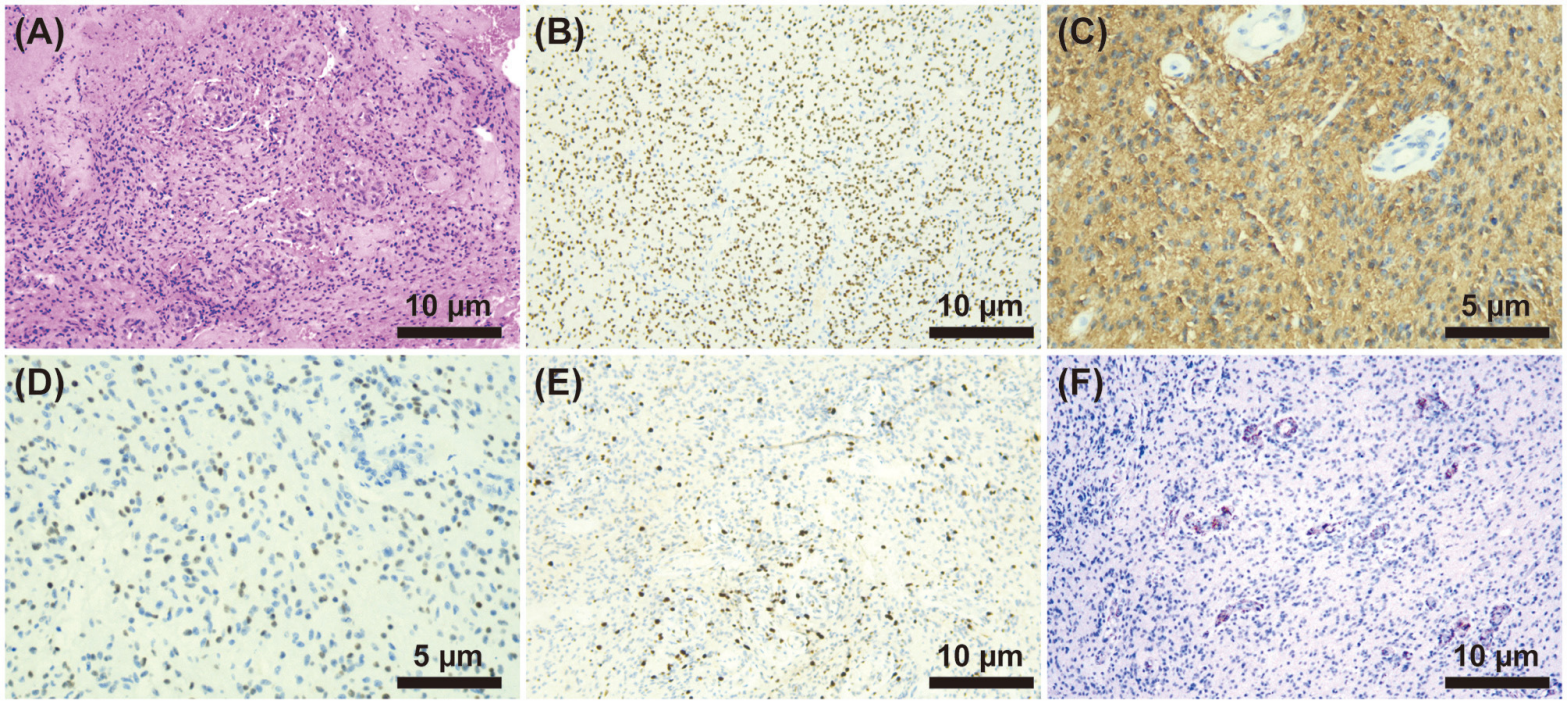
**(A)** HE staining (×200) showed diffuse growth of tumor cells, small and consistent tumor cells, visible microvascular proliferation, and occasional mitoses. **(B–F)** Immunohistochemistry. **(B)** H3K27M (+) (×200), **(C)** GFAP (+) (×400), **(D)** Oligo2 (+) (×400), **(E)** Ki-67(+) (×200), and **(F)** IDH-1(-) (×200).

On the 21st day of hospitalization, the patient's condition worsened. He was unconscious, his pupillary reflex disappeared, and he was diagnosed with a cerebral hernia. The patient was medicated to reduce intracranial pressure, and an emergency ventriculoperitoneal shunt was performed. The patient regained consciousness after surgery. On the 27th day, the thoracolumbar MRI scans were re-examined (Figure 1F), showing a significant increase in abnormal intramedullary signals at the thoracic (T) 12–lumbar (L) 1 vertebra level compared to that seen in the first scan. After that, the patient was transferred to the neurosurgery department. On the 35th day of admission, the patient underwent partial resection of space-occupying lesions in the spinal canal at T12–L1 of the vertebral body and spinal nerve adhesion release. The histopathological examination of the neuroepithelial tumor removed from the spinal tissue revealed a malignant tumor. The immunohistochemistry results showed GFAP (+), Oligo-2 (+), H3K27M (+), Ki-67 (+), IDH-1 (–), and EMA (–) (Figure 2). Combined with conventional pathological morphology and immunophenotype, the tumor was consistent with DMG, H3 K27-altered (WHO grade IV). The final diagnosis was DMG, H3 K27-altered. The patient was then transferred to the radiotherapy department and was recommended for local radiotherapy plus temozolomide chemotherapy. After radiotherapy, the patient's consciousness was better than before, and he could answer simple questions correctly. Abduction of both eyes was still limited, and pupillary reflex was found sensitive. His upper limb muscle strength improved, and the patient was discharged after his condition stabilized. No relevant disease assessment was performed during follow-up, and the patient died 6 months after discharge due to disease progression.

## Discussion

In this study, we report a case of DMG, H3 K27-altered in a 32-year-old man. The patient progressed rapidly and initially presented clinical characteristics of central nervous system infection. He was found to have a persistent headache and positive meningeal irritation. In our case, diffuse leptomeningeal and spinal dural enhancement was observed in the early stage of the disease, which is rare. It has been reported that diffuse or nodular leptomeningeal enhancement is seen in 12% of patients from the first imaging (7). According to Navarro et al. (8) leptomeningeal diffusion is usually considered a secondary sequela of DMG progression. It occurs in 17–56% of diffuse intrinsic pontine glioma (DIPG) cases in the pre-molecular era and indicates a very poor prognosis. The patient's lumbar puncture showed high intracranial pressure with high protein and low chlorine levels in the CSF. CSF cytology showed activated monocytes, but no tumor cells. The cerebrospinal fluid characteristics in our case were consistent with some literature reports (9, 10). However, there are only a handful of reports on the characteristics of cerebrospinal fluid from patients with DMG, H3 K27-altered.

The onset of this case was not typical. MRI and CSF showed manifestations similar to CNS infection, making early glioma diagnosis challenging. Diffuse enhancement of meninges and spinal meninges and the accompanied fever pointed toward central nervous system infection. In the subsequent analysis, we considered that the patient's fever may likely be due to pulmonary infection. The positive PPD and T-SPOT results for tuberculosis directed our diagnosis toward meningitis, especially tuberculous meningitis (TBM). The first symptom of TBM is fever, accompanied by symptoms of systemic tuberculosis, such as fatigue and night sweats. Cerebrospinal fluid manifestations include increased pressure, increased protein (often >100 mg/dL), decreased sugar and chloride levels, and increased white blood cell count (11). In the acute phase, the cerebrospinal fluid is mainly characterized by neutrophilia, and in the subacute phase, a mixed cytological response is observed (12). The main imaging manifestations are basal meningeal enhancement, hydrocephalus, and cerebral infarction (13). The positive rate of TBM diagnosis is not high, with 50–60% in CSF TBM culture (14) and 63–78% in next-generation sequencing (15, 16). Although our case had many similarities with tuberculous meningitis in cerebrospinal fluid manifestations and MRI, anti-tuberculous treatment was ineffective, and the white blood cell count was not high. In addition, there were activated monocytes in cerebrospinal fluid. Based on these clues, we turned our diagnosis toward the possibility of an intramedullary tumor.

DIPG is now classified as DMG, H3 K27-altered in the WHO Classification of Central Nervous System tumors (2). Common clinical symptoms include headache, dizziness, nausea, vomiting, blurred vision, increased intracranial pressure, limb motor and sensory disorders, and ataxia (17–19). The clinical symptoms are referable to the tumor location, with the most common midline locations being the thalamus, brainstem, and spinal cord. MRI findings also vary according to the site of tumor involvement. However, there are few reports of such cases in adults and within the spinal cord. Imaging features of DMG, H3 K27-altered are mostly heterogeneous and prone to changes such as cystic degeneration, necrosis, and hemorrhage, and the main body of the lesion usually showed hypointense on T1-weighted MR imaging and hyperintense on T2-weighted MR imaging (20). Lesions show enhancement and necrosis in 50% of patients with thalamic gliomas. The range of enhancement varies in 67% of pontine glioma cases, generally manifested as punctate enhancement or large sheet obvious enhancement (21). It should be noted that all tumors with H3K27M mutations are not diffuse midline gliomas. H3K27M mutations are also seen in gangliogliomas, pilocytic astrocytomas, and ependymal tumors. However, the H3K27M mutation is rare in these entities and may indicate a worse prognosis (22). The tumors that meet the following four criteria are defined as DMG, H3 K27-altered: (1) The lesions show diffuse infiltrative growth. (2) Lesions are located in the intracranial midline (such as the thalamus, brainstem, and spinal cord). (3) The histological appearance of the lesion is glioma. (4) H3K27M mutation exists (23). The tumor in our case met all these criteria, and hence, the diagnosis was confirmed as DMG, H3 K27-altered in the spinal cord at the level of about the T12–L1 vertebra.

In the family history, the patient's sister also died of glioma. However, no pathology analysis was done. At the epigenetic level, the K27M mutation, which substantially alters the post-transcriptional modification pattern of the H3 K27M locus, leads to H3K27 hypomethylation and affects transcriptional gene stability, which causes or promotes the development and progression of cancer (24). This histone modification has a certain familial nature, but it is vulnerable to a variety of effects such as the external environment. However, there is no evidence of familial tumor susceptibility to the disease, and further studies are still needed to explore the association in the future.

Current treatments for diffuse midline glioma with DMG, H3 K27-altered include surgery, radiotherapy, or combined chemotherapy, but the effect is poor, and the prognosis is very poor (19). The standard recommendation for radiation therapy for DMG, H3 K27-altered is 54 to 60 Gy for 6 weeks. According to some studies, radiotherapy delays the tumor progression for up to 3 months in 70–80% of patients (25). Temozolomide is generally used as a radiosensitizer that, in combination, enhances the effect of radiotherapy (26). However, a large number of studies have shown that temozolomide chemotherapy is very limited due to the lack of methylation of the MGMT promoter region in DMG and H3 K27-altered patients (27). Emerging diagnostic methods and therapeutic agents are currently continuously developing. At present, targeted therapies targeting specific biological markers are being further investigated. Drugs such as the selective dopamine receptor D2 antagonist ONC201 have been shown to have early clinical activity in this disease (25, 28). Liquid biopsy technologies are constantly improving to assess liquid plasma and CSF for extracted cell-free tumor DNA. The detection of cell-free H3K27M tumor DNA sequences in CSF may soon allow the diagnosis and tracking of these tumors over time (29). Therefore, in the future, these types of cases may be diagnosed with CSF analysis.

## Conclusion

The clinical manifestations and imaging features in our case lacked specificity, so it is a great challenge to render a definitive diagnosis. The clinical presentation was similar to CNS infection at the onset, with low white blood cell count and monocytes found in CSF cytology. The clues from our case can be used as a reference for the differential diagnosis of DMG, H3 K27-altered. PET may have been helpful in identifying the optimal biopsy location, but ultimately, histopathology was required to make the diagnosis. As observed from other case reports, the prognosis in our case was poor, and our patient had an overall survival of 8 months. There is a need for more studies to strengthen the knowledge and understanding of DMG, H3 K27-altered and make a clear diagnosis as soon as possible to prevent missed diagnosis and misdiagnosis.

## Data availability statement

The original contributions presented in the study are included in the article/supplementary material, further inquiries can be directed to the corresponding author.

## Ethics statement

Written informed consent was obtained from the individual(s) for the publication of any potentially identifiable images or data included in this article.

## Author contributions

XC: data collection and analysis, manuscript writing, and literature research. YL: manuscript writing and decision making of patient's diagnosis and treatment. HB: decision-making of patient's diagnosis and treatment, contributed ideas to the article, and supervision of the study. YZ: cytological examination and cerebrospinal fluid examination. JH: decision-making of patient's diagnosis and treatment. HL: assessment of the patient's electroencephalogram. All authors contributed to the article and approved the submitted version.
